# Management of non-tubal ectopic pregnancies analysis of a large tertiary center case series

**DOI:** 10.1007/s00404-023-07290-4

**Published:** 2023-12-11

**Authors:** E. Tremmel, T. Starrach, C. Buschmann, F. Trillsch, T. Kolben, S. Mahner, A. Burges, B. Kost, L. Ehmann, D. M. Burgmann

**Affiliations:** Department of Obstetrics and Gynecology, University Hospital, LMU Munich, Marchioninistraße 15, 81377 Munich, Germany

**Keywords:** Cesarean scar pregnancy, Cervical pregnancy, Cornual pregnancy, Methotrexate

## Abstract

**Purpose:**

Ectopic pregnancies include cesarean scar (CSP), cornual and cervical pregnancies. Various treatment modalities have been- described, but no standardized procedure has been defined so far. The aim of our analysis was to evaluate the diagnostics and treatment at the Department of Obstetrics and Gynecology, LMU University Hospital, Munich.

**Methods:**

In this retrospective, single-center analysis, 24 patients treated between 2015 and 2020 were analyzed. After verification of the diagnosis by imaging and HCG–analysis, the treatment was individually determined: therapy with methotrexate (MTX) locally with or without simultaneous systemic treatment, surgical treatment via curettage, excision with uterine reconstruction even hemi hysterectomy.

**Results:**

Ten patients presented with CSP, six with cervical and eight with cornual pregnancies. Median age was 34.6 years. CSP was treated with local MTX in six cases; five required additional treatment with systemic MTX or curettage. Primary curettage or surgery was performed in four cases. In cervical pregnancies the primary therapy with local MTX injection and systemic treatment was performed in 50%. One patient was treated with MTX and insertion of a Bakri balloon. Trachelectomy was required in one case. 50% of cornual pregnancies were treated with MTX locally and intramuscularly and 50% received surgery.

**Conclusion:**

Treatment strategies were based on the patient’s individual risk parameters. The results of this study show, that simultaneous treatment with local and systemic MTX had good outcomes and could avoid surgeries.

## What does this study add to the clinical work?

**Table:** 

Therapy decision of ectopic pregnancies should be made critically. Simultaneous injection of MTX locally and intramuscularly seems to be the successful therapeutic approach of our data.	

## Introduction

Ectopic pregnancy is defined by the implantation of a fertilized oocyte outside the uterine cavity and occurs in approximately 2% of all pregnancies [1]. We differentiate between tubal pregnancy, which is the most common localization and non-tubal pregnancy, which is rare with about 1%-3% [1]. Non-tubal pregnancies can be located inside the ovaries or cervix, interstitial or abdominal, or after previous cesarean section in the scar. Maternal mortality is about eight times higher in non-tubal pregnancies than in tubal [2]. Different risk factors for ectopic pregnancy exist: abnormal transport of the fertilized ovum within the fallopian tube, caused by interventions of reproductive medicine, uterine anomalies, previous ectopic pregnancy, pelvic inflammatory disease, presence of an intrauterine device, nicotine, and—particularly in CSP—a history of cesarean section (CS) [3]. Typical symptoms of an ectopic pregnancy are abdominal pain, vaginal bleeding, and/or abdominal swelling. Some ectopic pregnancies appear without any symptoms [4].

All-types of ectopic pregnancy can end in potentially life-threatening complications, for example rupture of the uterus wall or the tube with hemorrhagic shock or septic abortion and following sepsis [5]. Tubal and cornual pregnancies have a higher risk of severe hemorrhage because of the rich vascular network from anastomosis of the uterine and ovarian arteries [1]. Life-threatening bleeding in cornual pregnancy or CSP requires hysterectomy as the ultimate treatment option [6].

Non-tubal ectopic pregnancies represent a particular diagnostic and therapeutic challenge and are at a higher risk of complications with increased morbidity [7]. After CS, 6.1% of all ectopic pregnancies implant in the cesarean scar [8]. A cesarean scar pregnancy, also known as a uterine scar pregnancy, occurs when a fertilized egg implants and develops in the scar tissue of a previous cesarean section. The number of CSP is increasing due to the improvement of diagnostic techniques like ultrasound and the increased rate of CS in the last decade [8]. The CS rate increased up to 30% in developed countries, for example in the -USA [9]. According to the German Federal Statistical Office, this rate is quite in line with the German CS rate of 29.7% in 2020.

Cervical pregnancy is rare and occurs in only 0.1% of ectopic pregnancies. The most common symptom is vaginal bleeding [6].

An other special case of ectopic pregnancies is the cornual pregnancy. Cornual pregnancies account for 2.6% of all ectopic pregnancies and go along with a five times increased-risk of death [1]. A cornual pregnancy, also referred to as an interstitial pregnancy, is a rare form of ectopic pregnancy where the fertilized egg implants in the horn or lateral region of the uterus, outside the normal cavity, and it is associated with a high risk for severe hemorrhage, as mentioned above [1].The diagnosis of ectopic pregnancies is mainly made by transvaginal sonography [10]. If the location is not clear or if the infiltration of neighboring organs is suspected, magnetic resonance imaging can be considered [11]. This is helpful, especially in CSP, to analyze the thickness of the remaining uterus wall after CS [11].

In Germany, no guidelines regarding the treatment of ectopic pregnancies and ectopic pregnancies like CSP, cornual or cervical pregnancies exist. There are some guidelines concerning ectopic pregnancies, like the NHS Guidance “Guidelines on the diagnosis and management of ectopic pregnancy and pregnancy of unknown location”, the International Federation of Gynecology and Obstetrics (FIGO): ectopic pregnancy current management guidelines (2020) or the guideline of the American Society of maternal—fetal medicine or the guideline of the Society of Obstetricians and Gynaecologists of Canada (SOGC). But these mainly deal with tubal ectopic pregnancies. There are no recommendations concerning CSP, cervical, or cornual pregnancy. Pharmacological treatment options include systemic or local administration of methotrexate (MTX), chloride potassium, hyperosmolar glucose solution, prostaglandin, or a combination of the medications. The drugs are administered with laparoscopical assistance or are injected directly into the gestational sack under transabdominal or transvaginal ultrasound control [12]. The dosing for intramnial injection is 1 mg/kg body weight, also using a multidose scheme. In the single-dose scheme, 50 mg/qm is applied intramuscularly to the patient [13].

MTX is a common drug treatment in ectopic pregnancies as an alternative to surgical therapy. Seldom MTX used in pregnancies of unknown location (PUL). Requirement of it is that an appropriate intrauterine pregnancy is excluded, or when surgical treatment in abortion cannot be performed. Relative contraindications need to be considered such as βHCG > 4000, detection of an embryonic heartbeat, lactation, heterotope pregnancy, maternal comorbidities, allergies, missing compliance of the patient, renal insufficiency, moderate to severe anemia, leukopenia, or thrombocytopenia, liver disease or alcoholism and active peptic ulcer disease [14, 15]. In higher βHCG levels or with positive heartbeat, the rate of successful MTX treatment is decreasing. In Addition, before treatment, a complete blood count and a comprehensive metabolic panel should be checked [14]. Common side effects of MTX treatment must be considered like alopecia and elevated liver enzymes. During MTX treatment, patients should be informed to take folic acid supplement. Nonsteroidal anti-inflammatory drugs and sun exposure should be avoided [14]. Patients should receive counseling on the importance of refraining from becoming pregnant for a minimum of 3 months following their treatment [16].

Based on this information, the objective of this analysis was to conduct a retrospective evaluation of our treatment and success rate of ectopic pregnancy such as CSP, cervical, or cornual pregnancy and to contribute evidence for establishing a future standard.

## Materials and methods

### Study population

This is a retrospective analysis of a case series of 24 patients with diagnosed or suspected CSP, cervical pregnancy and cornual pregnancy referred to LMU University Hospital, Munich, between April 2015 and May 2020. Clinical and anamnestic data were retrieved from electronic clinical records of the patients. The following eligibility criteria had to be fulfilled: (1) the ultrasound images confirming the diagnosis were available and all structures were visible including embryo, yolk sac, exact localization, myometrium, both ovaries; (2) an adequate follow-up period was documented.

### Ethical approval

Our study was confirmed by the ethical committee of our University Hospital (Project- No. 23-0111, date: 21.03.2023). All procedures performed in the study were in accordance with the ethical standards of the Declaration of Helsinki.

### Diagnosis

The ectopic pregnancy was diagnosed by transvaginal ultrasound with the following criteria: empty uterine cavity, clear visibility of the ectopic pregnancy as well as the entire uterus with its fundic and cervical contour and ovarian/tubal area of both sides. In one case, MRI was performed in an external hospital to rule out a placenta increta in a cervical pregnancy.

### Treatment

Treatment modalities were as listed below:

- Ultrasound or laparoscopic guided injection (transvaginal or percutaneous) of 50 mg MTX intragestational [17].
- Systemic intramuscular injection of a single dose of 50 mg/m^2^ BSA (body surface area) MTX [17].
- Simultaneous local and systemic treatment including a and b.
- Dilation and curettage with or without vaginal prostaglandin priming of the cervix, with 400 μg prostaglandin.
- Primary laparotomy or laparoscopy and excision of the uterine scar pregnancy with reconstruction of the anterior uterus wall

The decision about which mode of therapy would be performed, was made after an interdisciplinary discussion. Patients got a detailed information about the various treatment options, including benefits and potential risks, and management of the procedure. Written informed consent was obtained. Patients remained hospitalized until effect of the treatment was proven by an adequate drop in serum βHCG.

Weekly determination of serum βHCG was performed in our hospital for an indefinite period of weeks and bimonthly until the hormone was below the detection limit.

### Follow-up

**Fig. 1:**
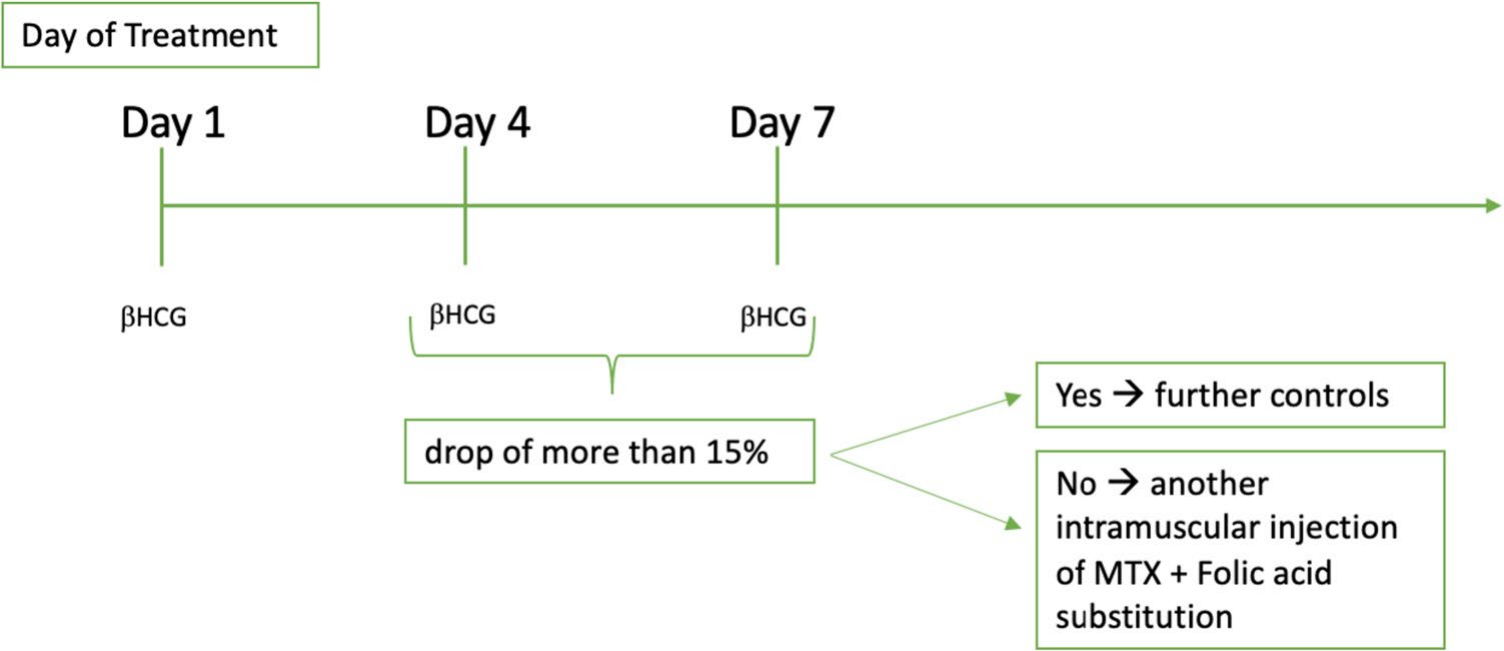
Scheme for follow-up after treatment with MTX, measuring the βHCG level in blood

The protocol for follow-up in the MTX-group was in accordance with the in-house standard for ectopic pregnancies, including the determination of serum βHCG, as shown in Fig. 1. Patients returned for a visit in our outpatient clinic.

### Statistical methods

The data were analyzed in an Excel spreadsheet, and statistical analysis was carried out at the nominal and ordinal levels. It has been calculated using Microsoft Excel and IBM SPSS Statistics 29.

## Results

Between April 2015 and May 2020, 24 patients were treated at LMU University Hospital, Munich with the diagnosis “other ectopic pregnancy” (ICD O00.8). Ten patients had CSP, eight had cervical pregnancy, six had cornual pregnancy. Major clinical characteristics in all patients are presented in Table 1.

**Table 1:** Overview of the number of pregnant women in the individual categories

The treated patient with other ectopic pregnancy (*n*)	24
Caesarean scar pregnancy (*n*)	10
Cervical pregnancy (*n*)	6
Cornual pregnancy (*n*)	8

### Caesarean Scar Pregnancy (*n* = 10) 

**Fig. 2:**
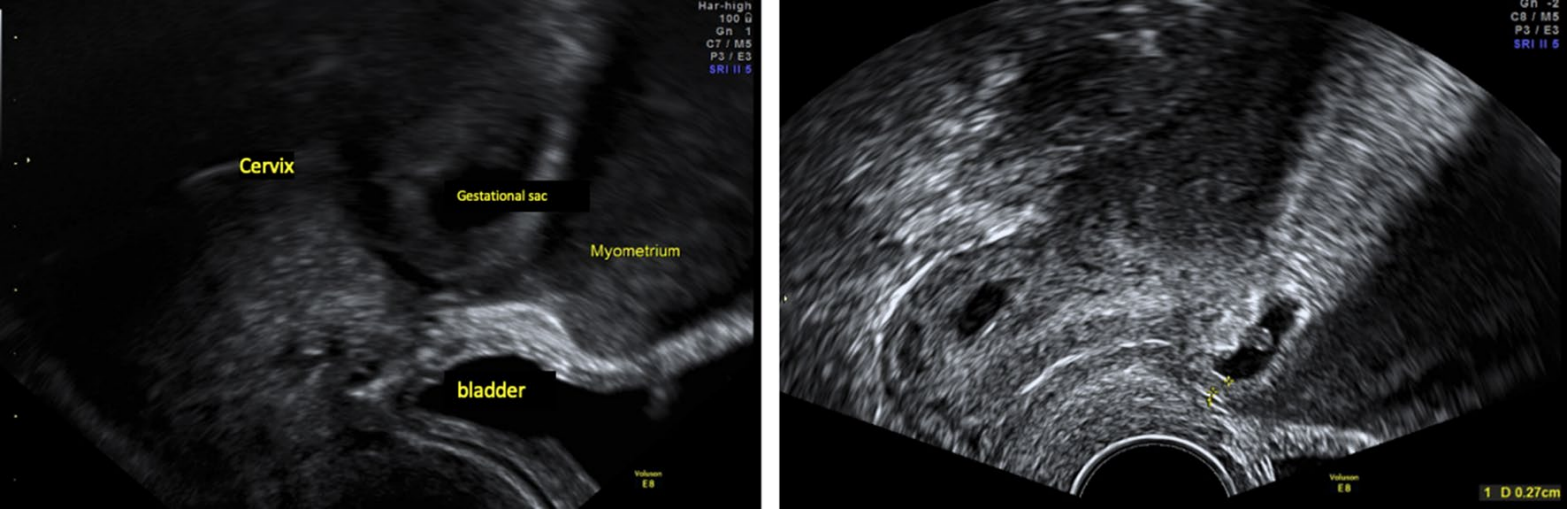
Ultrasound of CSP in a 42-year-old woman (G3 P2) in 6^2/7^ weeks of gestation, with one prior C-Section and a uterine wall remaining 0.27 cm

**Fig. 3:**
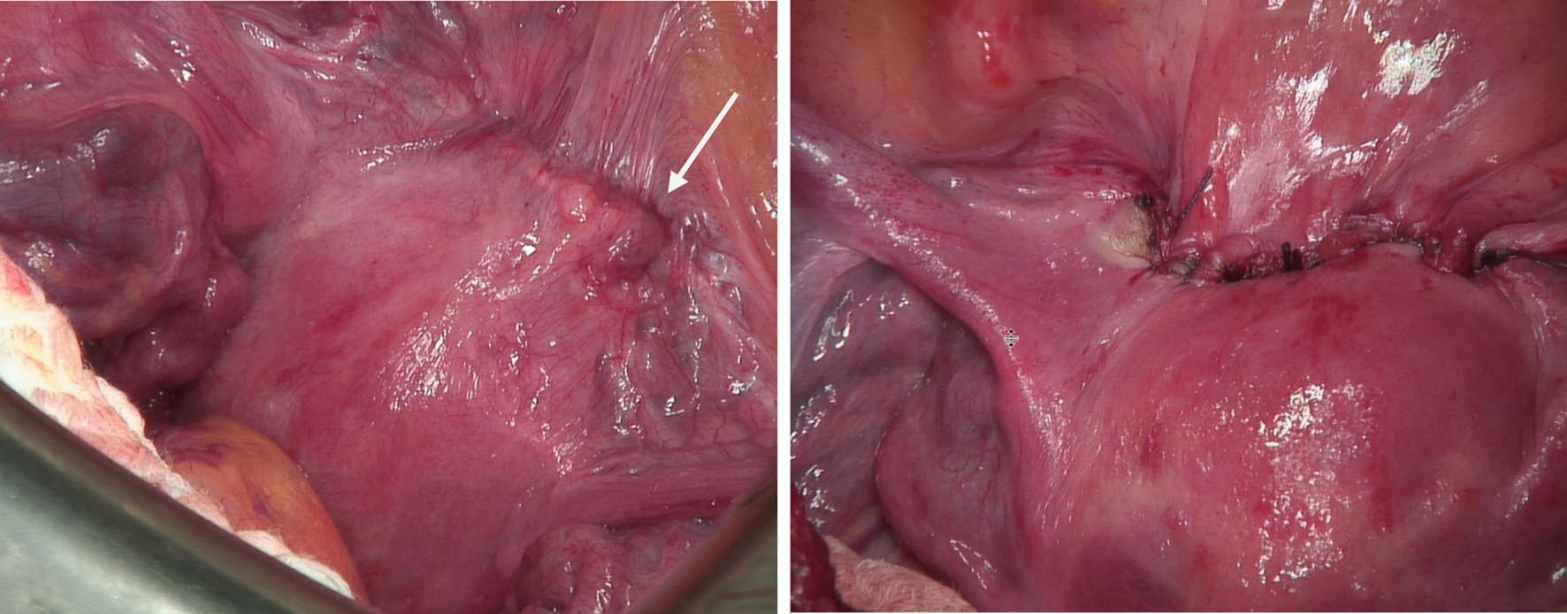
28-year-old woman (G4 P1) in 6^1/7^ weeks of gestation, with one prior C-section. CSP marked with white arrow before and after reconstruction

CSP occurred in ten patients. The patient’s age ranged from 28 to 42 years. Demographic data are shown in Table 2. Location of the pregnancy is shown in Figs. 2 and 3. Three out of ten cases were twin pregnancies, in two of these one fetus was in the scar and one in the uterine cavity. In all three cases, one fetus showed negative heart action independent of its location.

**Table 2:** Demographic characteristic in the three cohorts

	CSP (*n* = 10)	Cervical pregnancy (*n* = 8)	Cornual pregnancy (*n* = 6)
Age mean / range (years)	34.3/28–42	33.25/22–44	35.66/25–47
Gravidity mean/ range (*n*)	3/2–7	3/1–7	3/1–5
Previous cesareans (%)	100	50	33,33
More than one previous cesarean (%)	30	12.5	0
Previous curettage (%)	30	50	33.33
Previous therapy with MTX (%)	0	17	0
IVF/ICSI/egg donation (*n*)	2	1 Egg donation	1 Egg donation
Gestational age/total (weeks)	7/ 6–11	7/6–13	7
Initial serum βHCG value absolute range (Uml/mL)	9 255–102 000	706–93 820	3 772–63 391
Initial serum βHCG value mean (Uml/mL)	33.759	33.114	29.603
Embryonic structure present (*n*)	5	1	1
Positive heart action (*n*)	5	2	1
Size of amniotic sac (mm)	21.83 (16–40)	20(12–23)	18.33 (9–25)
Twin pregnancy (*n*)	3	1	0
Hospitalization (%)	100	100	100
Median duration of stay (days)	6.1	6.5	5.5
Maximum duration of stay (days)	13	14	13
Transfusion (*n*)	1	1	0
Following pregnancy reported (*n*)	2	1	1

Six patients (*n* = 6) initially received a local injection of MTX into the amniotic sac as primary therapy, either guided by transvaginal ultrasound (*n* = 4/6) or guided by abdominal ultrasound (*n* = 2/6), those two injections were performed percutaneously (*n* = 2/6). In four of those six cases (*n* = 4/6), a simultaneous systemic MTX dose via intramuscular injection (i.m.) was given and in two of those six cases (*n* = 2/6), additional curettage was performed due to a relevant hemorrhage.

Two patients (*n* = 2) with CSP received primarily curettage, one needed a secondary reconstruction of the scar.

Two other patients (*n* = 2) received primary laparotomy: in one case because of a thin uterine wall (< 2 mm) in the diagnostic, which was verified intraoperatively as a perforation. In the other patient showed intraabdominal bleeding by diagnosis, which was confirmed as a ruptured uterine scar on MRI. In both cases, the uterine wall was reconstructed after the excision of the scar and pregnancy (Fig. 2).

During follow-up period, four patients expressed no whish for a further pregnancy. Two patients became pregnant again, and in both cases, sonography revealed the presence of at least a minor defect in the myometrium once more. To avoid uterine rupture caesarean section was perfomed as delivery mode (Fig. 3).

### Cervical pregnancy (*n* = 8)

Eight patients were diagnosed with cervical pregnancy (*n* = 8). The patient’s age ranged from 22 to 44 years. Demographic data are shown in Table 2. The documented average size of the amniotic sac was 20 mm. Transfusion was required in one patient with relevant vaginal bleeding. In this case, the performed MRI showed a placenta increta.

50% (*n* = 4/8) were treated by a primary MTX therapy locally administered. Two of these patients had an additional simultaneous intramuscular MTX injection. Two patients received repeated local and intramuscular injections. As a side information, one patient had a local infection after systemic treatment with MTX.

In three cases, a Bakri balloon was inserted—in one case as a primary treatment, in one after curettage, and in the third one after the locally administered MTX.

The patient with vaginal bleeding mentioned above had a cervical pregnancy in the 12+2 gestational week of gestation with placenta increta. The patient was presented primarily to another hospital and was transferred to our clinic because of vaginal bleeding at diagnosis and a minimal hemoglobin level of 7.6 g/dl. Therapy included trachelectomy via transverse laparotomy by placenta increta due to emergency situation. Intraoperative cerclage sutures were performed prophylactically. This patient developed stenosis of the remaining cervical rest, which was corrected by conization 2 years later for the reason of a persisting wish to get pregnant. During follow up, one successful pregnancy could be observed in a case after MTX treatment.

The patient with trachelectomy was counseled in the department of reproductive medicine, but no following intervention or pregnancy was documented. One patient was not able to conceive a pregnancy even after laparoscopy performed as part of fertility treatment.

### Cornual pregnancy (*n* = 6)

**Fig. 4:**
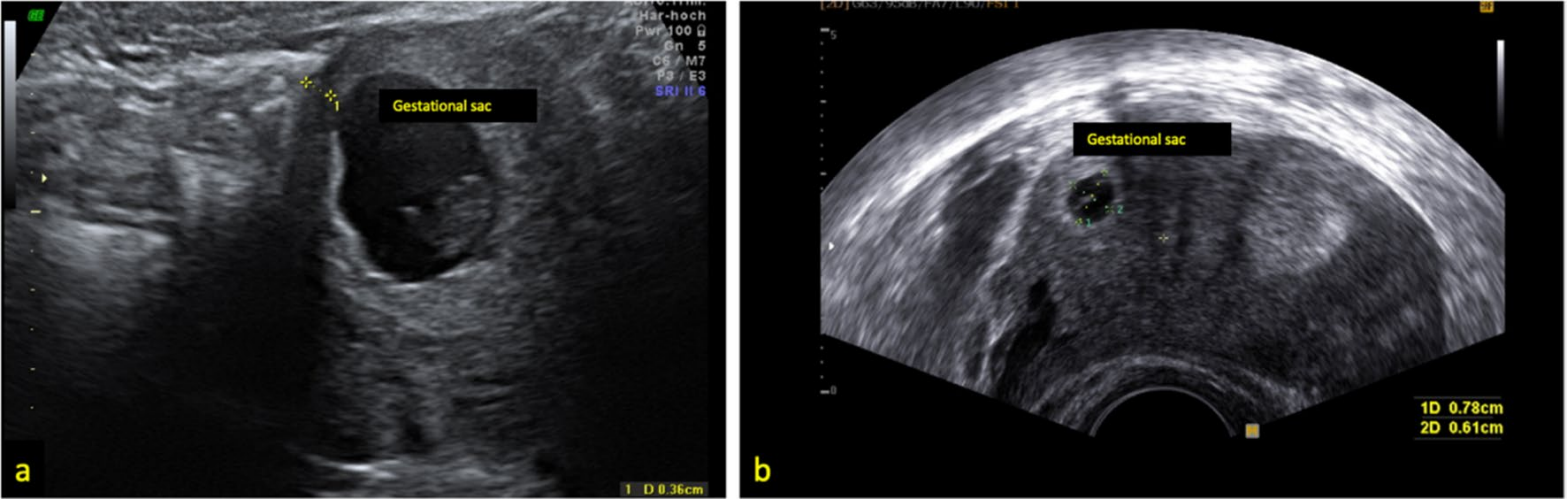
a + b examples for cornual pregnancy. **a** 32y G5 P2 in 9+6 weeks of gestational age, myometrium remaining 0.36 cm. therapy: MTX local and i.m.; **b** 33y G5 P3 in 4+4 weeks of gestational age, condition after C-section and dilation and curettage, size of gestational sac 0.78 × 0.61 cm (GS—gestational sac)

Six patients were diagnosed with cornual pregnancy (*n* = 6). This is the smallest subgroup in our retrospective analysis. Location of this pregnancy is shown in Fig. 4. The patient’s age ranged from 25 to 47 years. Demographic data are shown in Table 2. This group included one pregnancy after oocyte donation. Only one patient presented with an embryonic structure and a positive heart action in the ultrasound. The amniotic sac was between 9 and 25 mm. One of the cornual pregnancies showed a location in a rudimentary uterine horn of the uterus bicornis unicollis. An other patient showed a trophoblast invasion into the back wall of the uterus.

In this group, local MTX therapy was combined with intramuscular MTX therapy in four cases. Two cases received injections via laparoscopy, two transvaginal. One patient required three additional MTX injections systemically after local treatment. This patient developed a mucositis as a side effect.

Two patients needed extended surgical therapy: one received primary curettage of the pregnancy in the uterine horn. In this case, the pregnancy was in the stump of the.

remaining tube after salpingectomy which was performed due to a previous ectopic pregnancy 3 years prior. During curettage, the uterine wall was perforated. As a result, laparoscopy had to be performed to deal with the complication and and local MTX application and additional tube sterilization.

The other patient who needed surgery required a hemi-hysterectomy. Because of the location of the pregnancy in the left rudimentary uterine horn of a uterus bicornis unicollis, this had to be removed. One patient out of the medical treatment group needed to be controlled after MTX injection for 71 days in our clinic, which is above average.

In the follow-up, one pregnancy after the hemi- hysterectomy was documented. In Tables 3 and 4 you can see an overview of treatment modalities an complications.

**Table 3:** Overview of primary treatment modalities according to ectopic pregnancy

	MTX locally	MTX locally+systemically	Surgery	MTX systemically+surgery	Other combinations
CSP (*n*)	1	5	4	1	–
Cervical pregnancy (*n*)	–	4	2	–	3
Cornual pregnancy (*n*)	–	4	2	–	–
Total (*n*)	1	13	8	1	1

**Table 4:** Overview of complications, blood loss, HCG level behavior and hospitalization according to treatment modalities

	Complications	Blood loss	Decreasing of HCG	Hospitalization(d)
MTX locally (*n* = 1)	Later dehiscence, reconstruction necessary	Not relevant	Adequate	3
MTX locally+systemically (*n* = 13)	1 Mucositis after 4 systemic applications	Not relevant	2 Times βHCG controls > 60 days till adequate decrease	2–13
Surgery (*n* = 8)	1. Perforation during curettage in Cornual Pregnancy 2. Cervical stenosis after trachelectomy in Cervical Pregnancy 3. HCG persistence after curettage in CSP, secondary reconstruction	1. Not relevant 2. Transfusion 3. Transfusion	1. Adequate 2. Adequate 3. Secondary adequate	8–14
MTX+surgery	–	Not relevant	Adequate	2
Others	Bakri catheter additionally to MTX locally or systemically or curettage by cervical pregnancy	Not relevant	Adequate	2–7

## Discussion

Non-tubal ectopic pregnancies are rare but challenging in diagnosis and treatment. They have a high risk of life-threatening complications. To date, no special treatment guidelines for non-tubal ectopic pregnancies exist. However, data are shown in the literature with various medical, surgical, and interventional treatment modalities. The outcome of non-tubal ectopic pregnancies depends on a correct diagnosis and appropriate treatment. The most common treatments in literature include MTX, curettage, hysteroscopy, transvaginal resection, uterine artery embolization, laparoscopy, and high-intensity ultrasound [18–22].

In our cohort of ectopic pregnancies, we differentiated three cohorts of ectopic localizations: cesarean scar pregnancy, cervical pregnancy, and cornual pregnancy. As shown in Table 1, there was no difference in the demographic characteristics.

### Summary of findings

In our single-center retrospective analysis, we evaluated the cases of non-tubal ectopic pregnancies treated in our hospital to add evidence to provide a basis for a future standard of care.

**Fig. 5:**
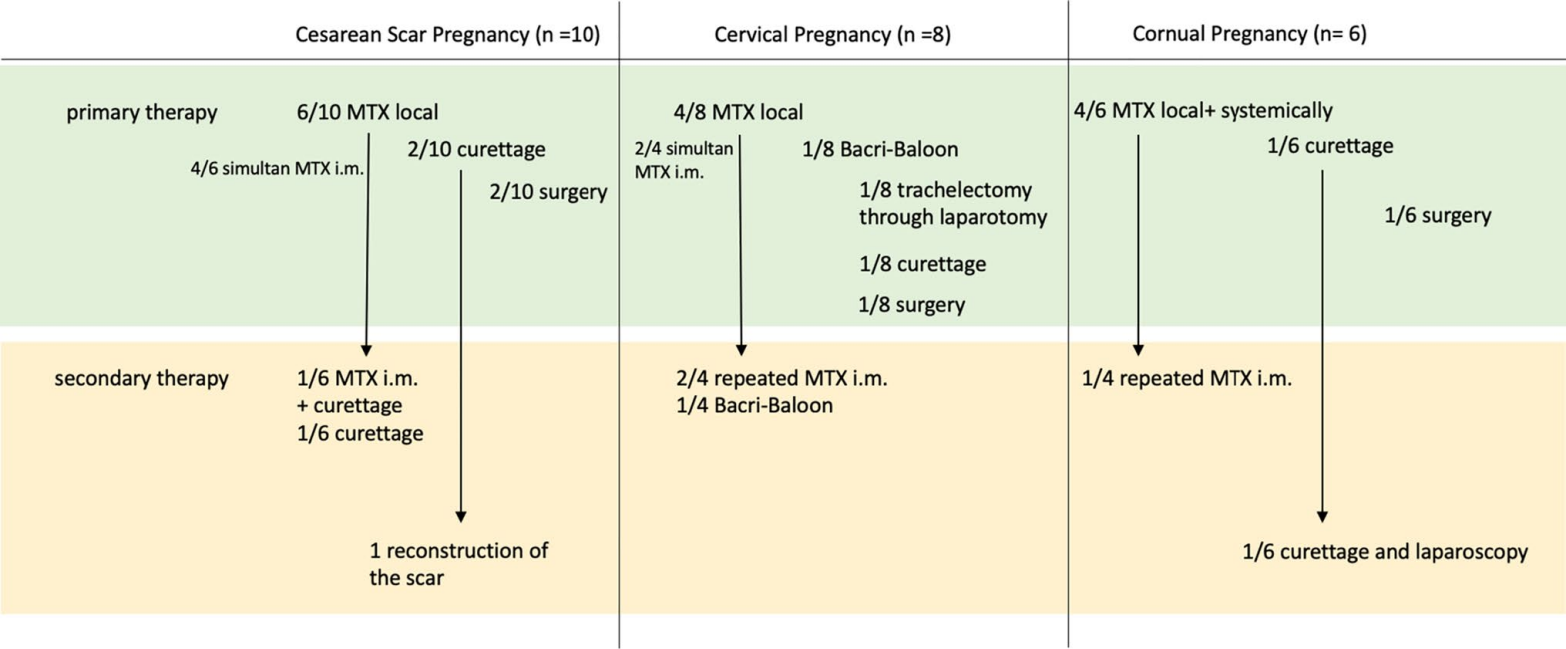
Scheme of therapy modalities in our retrospective analysis

Our data show an individual approach depending on the diagnostic outcome and experience. After a primary systemic therapy with MTX, 50–75% of the patients over all groups needed any secondary therapy (MTX i.m. or Bakri-balloon). Especially those without simultaneous local and systemic MTX treatment. In CSP group, 50% needed a secondary surgical therapy. In the case of CSP, a future wish to conceive highly relevant. Regarding a future pregnancy surgical excision, and reconstruction is the safest treatment option for these patients. Cornual pregnancy and cervical pregnancy did not require any secondary surgical treatment after primary MTX. In the group of cervical pregnancy and cornual pregnancy, just one case each required primary surgery (trachelectomy or hemihysterectomy). In our analysis, simultaneous local and systemic MTX therapy appeared to be the most successful treatment. Therapy modalities are shown in Fig. 5. Up to now, 4 of 25 patients successfully got pregnant again.

Overall, complications and side effects were minor. Just one of our patients developed mucositis, after repeated treatment with MTX. Blood transfusion was only indicated for one patient due to bleeding (Fig. 5).

### Implications for clinical practice

An individual consideration of the patient’s history, risk factors, family planning, and current circumstances should be considered during the process of decision making to find an optimal and safe treatment. However, primarily combined treatment modalities, e.g., local and systemic MTX or MTX and surgery, can help to reduce hemorrhage, the risk of further surgery, and result in an adequate treatment success. In some cases, a multimodaltreatment concept may be necessary to reduce the risk for the patient and optimize the outcome. Also, the patient’s compliance and social factors (e.g., work and family commitments, lack of transportation) [14] must be considered, especially in the outpatient sector since controls must be performed following a strict algorithm. In our cohort, inpatient treatment was preferred because of a lack of experience and standardized recommendations for treating non-tubal ectopic pregnancies. So far, MTX is the only successful conservative treatment. Several MTX protocols for ectopic pregnancies can be found in the literature. Over all groups, we followed our therapeutic regime with MTX 50 mg/m^2^ BSA.

Surgical treatment is indicated if there is a rupture of the uterine scar or a high risk for uterine rupture, especially in CSP or cornual pregnancy. In some cases, surgical treatment can be safer, e.g., regarding a future pregnancy, as the primary excision of the cesarean scar including the reconstruction of the uterine wall can prevent severe hemorrhage or secondary complications. We recommend laparotomy, because it is important to remove the pregnancy in total and to give the best possibility to reconstruct for example the cesarean scar. This is not a new technique, but it’s our experience we want to share, compared to other studies, who performed laparoscopies. We would like to point out the importance of a guideline concerning this topic.

Concerning CSP, systemic drug treatment was preferred but in 30% of cases surgery became necessary. Petersen et al. showed, in their systematic review, all kinds of treatments of CSP and the outcome of over 2000 CSP [18]. In cases with CSP, Timor-Tritsch et al. described a splitting of the locally administered MTX. Half of the dose was injected into the fetus and the other half into the placenta site resulting in a reasonable success rate [19]. In addition, locally applied MTX or surgical approaches like surgical aspiration, curettage, hysteroscopy, transvaginal resection, and laparoscopy, and interventional techniques like uterine artery embolization and high-intensity ultrasound were mentioned as therapy options [18]. As in our cohort, primary laparotomy was only performed when necessary due to imminent risk of uterine rupture or bleeding. In the study of Petersen, a secondary treatment was required in 25% of the cases [19], whereas the rate for secondary treatment in our analysis was about 30%. Petersen et al. did not find any correlation or consensus on a treatment strategy in relation to special conditions of CSP, such as thickness of myometrium or relation to bladder. Instead of curettage, surgical aspiration should be considered as a possible gentle option. All in all our finding agrees with the results of other research groups.

Concerning cervical pregnancies, acceptable results could be achieved in 87.5% of our cases with primary MTX treatment with or without secondary treatment independent of size or localization. Other studies showed further approaches and methods. Imai et al. investigated hysteroscopic transcervical resection with a temporarily laparoscopic clipping of both uterine arteries (LUA clipping-TCR) as an effective minimally invasive treatment for the management. They showed that this is an effective management regarding fertility preservation in case of cervical pregnancy [20]. Hosni et al. described several effective conservative managements using MTX and additional potassium chloride. Furthermore, they used local hydrogen peroxide or Mifepristone [21]. Their review further pointed out that the treatment should be performed in a specialized center [21]. We agree that treatment of such ectopic pregnancies should be bundled in clinical centers to optimize the outcome for the patients and gain more experience. LUA clipping TCR seems to be an interesting alternative or additional treatment option. Here a guideline that specifies which procedure is when indicated would be helpful.

Cornual pregnancies showed a good outcome after conservative treatment (primary and secondary) without any need of surgery. Concerning cornual pregnancy, Mao et al. reported that the risk of persistent ectopic pregnancy is higher in multipara, if the maximum diameter of the lesion is smaller than 1.5 cm and if the dilation and curettage is not assisted by ultrasound guidance. The group of Mao recommend cornostomy or cornectomy for patients with cornual pregnancy. If curettage is performed or the location of pregnancy can be better reached from the abdomen, laparoscopic monitoring or injection from the abdomen should be performed. If a positive result of surgical therapy is to be expected, this should be preferred They do not recommend using MTX as a routine method at all [22]. We see the difficulty choosing good criteria to decide whether surgery or medical treatment will be successful. Therefore, drug therapy should be attempted in our opinion first. If surgery is the primary therapy, localization should be known exactly, especially before curettage. As we have noticed the high risk of iatrogenic perforation of the uterus. Stationary monitoring seems reasonable. Because of the small number of cases, no correlation of the size of the lesion and the therapeutic approach could be found.

Finally, we evaluated our cases of ectopic pregnancies in rare sites to establish a standardized treatment. In our cohort of 24 cases, we determined that a combination of different therapy modes based on systemically administered MTX led to an acceptable outcome and could prevent further surgery. Simultaneous intramuscular and local injection of MTX was applied in most of the patients under MTX treatment. Because of the small sample size due to these rare diagnosis, no significant correlations could be found. This is a weak point in our single-center study. However, βHCG blood levels and the size of the amniotic sac could be assumed to be important factors in decision making regarding therapy and outcome concerning our study and based on literature research.

In conclusion, treatment strategies were based on the individual risk and history of the patient, localization, and size of the ectopic pregnancy as well as βHCG levels. Exact ultrasound diagnostics are mandatory. Treatment modalities, and individual risk of the patient should be considered. Contraindications should be considered before starting drug treatment. The simultaneous injection of MTX locally and intramuscularly is a successful therapeutic approach of our data, which shows the main point of this study and to avoid surgery. A repeated application or secondary surgery can become necessary to optimize the outcome.

## Data Availability

The data used to support the findings of this study are included within the article.
